# *“It was difficult to offer same day results”*: evaluation of community-based point-of-care testing for sexually transmitted infections among youth using the GeneXpert platform in Zimbabwe

**DOI:** 10.1186/s12913-022-07557-7

**Published:** 2022-02-10

**Authors:** Kevin Martin, Chido Dziva Chikwari, Constance R. S. Mackworth-Young, Mutsawashe Chisenga, Tsitsi Bandason, Ethel Dauya, Ioana D. Olaru, Suzanna C. Francis, Constancia Mavodza, Portia Nzombe, Rangarirayi Nyamwanza, Fadzanai Hove, Maureen Tshuma, Anna Machiha, Katharina Kranzer, Rashida A. Ferrand

**Affiliations:** 1 Department of Global Health and Infection, Brighton and Sussex Medical School, Brighton, UK; 2 Clinical Research Department, London School of Hygiene & Tropical Medicine, London, WC1E 7HT UK; 3 Biomedical Research and Training Institute, Harare, Zimbabwe; 4 Department of Infectious Disease Epidemiology, London School of Hygiene & Tropical Medicine, London, UK; 5 Department of Public Health and Policy, London School of Hygiene & Tropical Medicine, London, UK; 6 AIDS and TB Unit, Ministry of Health and Child Care, Harare, Zimbabwe; 7 Division of Infectious and Tropical Medicine, Medical Centre of the University of Munich, Munich, Germany

**Keywords:** Sexually transmitted infections, chlamydia, Gonorrhoea, Point-of-care tests, Youth, Africa, Community-based settings

## Abstract

**Background:**

Point-of-care testing for sexually transmitted infections (STIs) may improve diagnosis and treatment of STIs in low- and middle-income counties. We explored the facilitators and barriers to point-of-care testing for *Chlamydia trachomatis* (CT) and *Neisseria gonorrhoea* (NG) for youth in community-based settings in Zimbabwe.

**Methods:**

This study was nested within a cluster randomised trial of community-based delivery of integrated HIV and sexual and reproductive health services for youth aged 16 to 24 years. On-site CT/NG testing on urine samples using the Xpert® CT/NG test was piloted in four intervention clusters, with testing performed by service providers. On-site testing was defined as sample processing on the same day and site as sample collection. Outcomes included proportion of tests processed on-site, time between sample collection and collection of results, and proportion of clients receiving treatment. In-depth interviews were conducted with nine service providers and three staff members providing study co-ordination or laboratory support to explore facilitators and barriers to providing on-site CT/NG testing.

**Results:**

Of 847 Xpert tests, 296 (35.0%) were performed on-site. Of these, 61 (20.6%) were positive for CT/NG; one (1.6%) received same day aetiological treatment; 33 (54.1%) presented later for treatment; and 5 (8.2%) were treated as a part of syndromic management. There was no difference in the proportion of clients who were treated whether their sample was processed on or off-site (64% (39/61) vs 60% (66/110); *p* = 0.61). The median (IQR) number of days between sample collection and collection of positive results was 14 (7–35) and 14 (7–52.5) for samples processed on and off-site, respectively,

The interviews revealed four themes related to the provision of on-site testing associated with the i) diagnostic device ii) environment, iii) provider, and iv) clients. Some of the specific barriers identified included insufficient testing capacity, inadequate space, as well as reluctance of clients to wait for their results.

**Conclusions:**

In addition to research to optimise the implementation of point-of-care tests for STIs in resource-limited settings, the development of new platforms to reduce analytic time will be necessary to scale up STI testing and reduce the attrition between testing and treatment.

**Trial registration:**

Registered in clinical trials.gov (NCT03719521).

**Supplementary Information:**

The online version contains supplementary material available at 10.1186/s12913-022-07557-7.

## Background

The World Health Organization (WHO) African Region was estimated in 2016 to have the highest prevalence for chlamydia (CT) in men, and gonorrhoea (NG) and trichomoniasis in both men and women [1]. Among clinic and community populations in Southern and Eastern Africa, risk is noted to be particularly high for young women, with higher prevalence than in older age groups [2]. Furthermore, a 2021 study amongst youth in community settings in Harare, Zimbabwe revealed CT and/or NG prevalence of 18.2 and 10.0% amongst women and men, respectively [3]. Importantly, testing in this study was not targeted at those with high-risk behaviour or symptoms, and uptake of testing was 33.3% [3]. Sexually transmitted infections (STIs) are associated with poor reproductive health outcomes, and an increased risk of HIV transmission [4]. Specifically, CT and NG infection can cause pelvic inflammatory disease in women with possible sequelae including infertility, risk of ectopic pregnancies and chronic pelvic pain [1].

The WHO has recommended syndromic management of STIs in resource-limited settings since 1991, predominantly due to a lack of high quality, affordable diagnostics [5]. However, the majority of infections are asymptomatic and will not be identified through syndromic management, leading to onward transmission of these infections [6]. An alternative option is laboratory testing away from the point of care. However, this requires multiple steps between sample collection and treatment of a positive case, including a (correctly labelled) sample being sent to the laboratory, testing the sample, producing the result, sending the result back to the referring clinic, informing the patient, and the patient returning for treatment. Pre-treatment loss-to-follow-up (LTFU) in low-income countries is well documented for syphilis and other diseases including tuberculosis [7, 8]. One strategy to reduce LTFU is to provide same day results through on-site STI testing. For syphilis, the advent of inexpensive, accurate and rapid lateral flow point-of-care serological tests has enabled substantial increases in syphilis screening particularly in antenatal care in resource limited settings [9]. In addition to higher screening rates, point-of-care testing for syphilis has also been associated with higher numbers of syphilis cases detected, higher treatment rates for both pregnant women and their partners, and lower incidence of congenital syphilis [10]. On-site testing with same-day results can also enable screening for marginalised populations, and those living in remote regions, for whom access to clinical services may be difficult [11–15]. Mobile testing units offering HIV and syphilis screening have been shown to reach higher risk populations than clinic-based testing [16]. For point-of-care tests for CT/NG, the evidence base is more limited. However, the WISH study demonstrated that sensitivity and specificity of the Xpert® CT/NG was superior to syndromic management in women at risk of STIs in Rwanda, even when only testing women who had at least one of a number of risk factors [17].

The WHO Sexually Transmitted Diseases Diagnostics Initiative developed a set of criteria as a benchmark for tests to meet STI control needs, recommending them to be affordable, sensitive, specific, user-friendly, rapid, robust, equipment-free and deliverable to end-users (The “ASSURED” criteria) [18]. Crucially, lateral flow assays for CT/NG are not accurate and molecular tests are required, which have been laboratory-based until recently. There is now a pipeline of closed cartridge molecular tests that can be performed near point of care [19]. The Xpert® CT/NG was the first test to be approved by the United States Food and Drug Administration and CE marked for CT/NG testing. It is run on a platform of a two, four, or sixteen-module machine, requires an uninterrupted power supply and has an analytic time of ninety minutes. A meta-analysis of studies testing urine, vaginal, endocervical, anorectal, and pharyngeal samples for CT/NG revealed pooled sensitivities and specificities of 94 and 99% for CT, respectively, and 95 and 100% for NG, respectively [20]. Importantly, due to its role in tuberculosis diagnostics, the GeneXpert platform is widely available in many resource-limited settings. It has also been reported that many countries are underutilising the GeneXpert machines they have, with opportunities missed to reduce redundancy and save costs through its use as a multi-disease platform [21].

The Xpert CT/NG has been trialled as a point-of-care platform in resource-limited settings in antenatal care [22–25], primary healthcare [26, 27], and in high-risk individuals in research settings as part of diagnostic evaluations [17, 28]. In these settings, high levels of both acceptability and feasibility were reported. However, in addition to being at high risk of STIs, young people face additional barriers to accessing sexual and reproductive health services, including service availability, acceptability, and stigma [29]. These barriers must be considered and addressed in the development of STI control strategies. Lessons must be learnt from HIV, where multiple diverse testing strategies have been demonstrated to be necessary, with a one-size-fits-all approach to testing being insufficient [30, 31]. Similarly, we must consider alternative strategies for delivery of STI services outside of the traditional healthcare model. This study aims to provide evidence of such a testing strategy. We investigated the feasibility of and the barriers and facilitators to the implementation of on-site CT/NG testing using the GeneXpert platform in community-based settings in Zimbabwe.

## Methods

### Setting

This study was nested within the CHIEDZA trial (“Community based interventions to improve HIV outcomes in youth: a cluster randomised trial in Zimbabwe”), a cluster randomised control trial (registered in clinical trials.gov: NCT03719521) investigating the impact of a community-based package of integrated HIV and sexual and reproductive health (SRH) services for youth on HIV and other health outcomes [32]. The two-arm trial is being conducted in 24 clusters in three provinces (Harare, Mashonaland East and Bulawayo), with eight clusters in each province randomised 1:1 to intervention or standard care (routine, existing services) arms. A cluster is a geographically demarcated area containing a primary health care clinic and a community centre from which services are delivered.

Individuals aged 16 to 24 years living within an intervention cluster are eligible to receive a package of services including HIV testing, HIV treatment and adherence support, contraception, pregnancy testing, syndromic management of STIs, menstrual health information and products, risk reduction counselling and condoms, and general health counselling. In each cluster, services are delivered once weekly (on the same day each week) by a team of nurses, community health workers (CHWs), youth workers, and a counsellor. Services are provided in tents (“booths”) that are set up in non-clinical community halls or youth centres. The intervention was co-designed with youth, aiming for youth-friendly, non-judgemental, and confidential services. The services are provided from 10 am to 5 pm operating as a walk-in service with no prior appointment required and offered free of cost.

### Nested CT/NG study

The nested CT/NG study was conducted as a pilot to offer point-of-care CT/NG testing in the four intervention clusters in Bulawayo which is the second largest city in Zimbabwe.

### Implementation of GeneXpert platform in a community setting

**Fig. 1:**
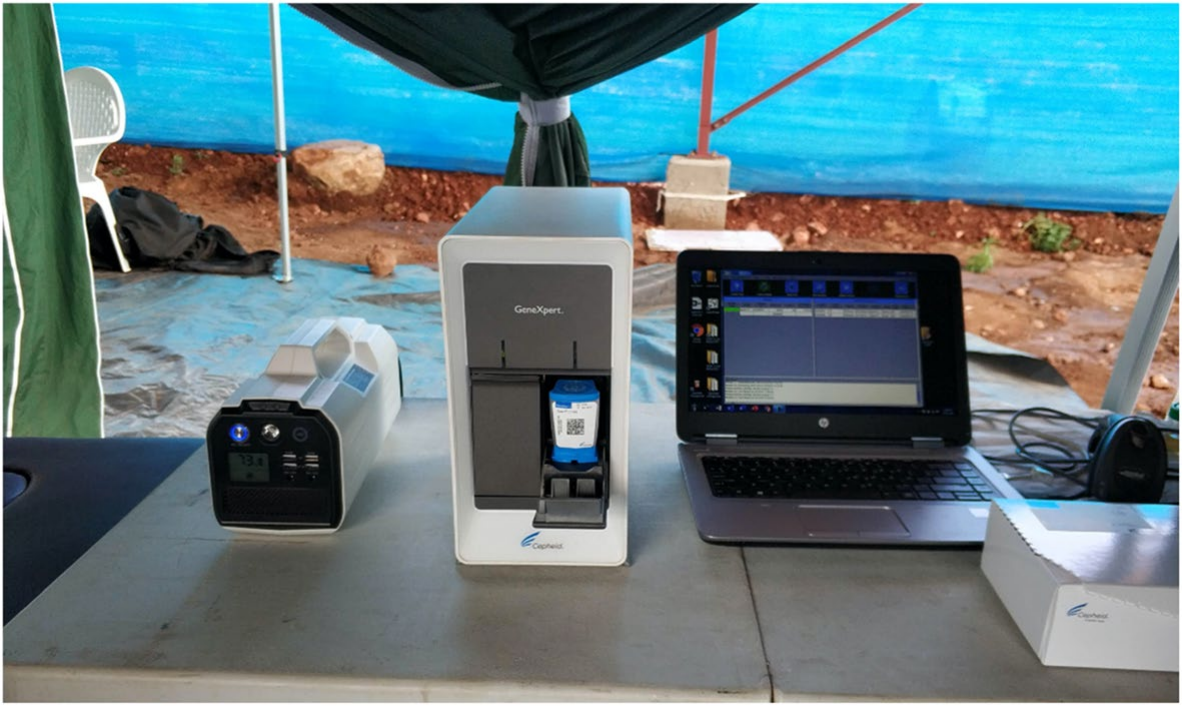
Photograph of testing set-up

A two-cartridge GeneXpert machine was set up daily in each cluster from 25th November 2019 to 3rd February 2020, before being replaced with a four-cartridge machine from 4th February 2020 to 18th March 2020. Due to unavailability of power in the field or frequent power shortages, the GeneXpert machine was operated using a rechargeable powerpack (SOLARGEN-500; Model number PHD-500SG) which provided an uninterrupted power supply (UPS) for 8 h in the field (Fig. 1). The GeneXpert machine and powerpack were returned to the research office every evening, where the powerpack was recharged using mains electricity. A diesel generator was also available to charge the powerpack on-site if necessary and if mains electricity was not available. Temperature logs were maintained for the storage of GeneXpert cartridges at the team’s headquarters, with storeroom temperature documented twice per day, at 8 am and at midday***.***

The nine providers were a combination of nurses, CHWs, youth workers or counsellors, all of whom were trained on the standard operating procedures (SOPs) developed by the study team based on the manufacturer’s instructions. The CHIEDZA providers were trained to use the machine during a one-day course on 22nd November 2019. A further refresher training session was provided on 4th February 2020. Of the providers, only one (a CHW) had a laboratory background (BSc in Microbiology) but all providers were on a daily rota to run the machine. Sample preparation time took roughly 5 min and analytic time was 90 min. A maximum of eight or sixteen samples could be run on-site each day using the two- and four-cartridge device, respectively. As CHIEDZA services did not start until 10 am, it was possible for two or four samples to be processed the following morning at the research office. As CHIEDZA services run Monday to Thursday; Fridays and the weekends were used to catch up with testing at the research office. Additional samples were also sent in a cool box via courier to the central laboratory in Harare for processing during periods of particularly high demand. When samples were sent to Harare for processing, results were sent back to Bulawayo via a combination of e-mail and WhatsApp messaging.

### CT/NG testing procedures

All individuals accessing CHIEDZA services in Bulawayo province were non-selectively offered confidential testing for CT and NG, regardless of whether they had symptoms. Those who accepted testing provided a first-catch urine sample. On-site testing was defined as sample processing on the same day and at the same site as sample collection. Off-site testing involved testing at any other date or location. Clients whose samples were processed on site could wait to collect their results on the same day. If clients could not wait for their results or testing was carried out off-site, clients were asked to return for their result the following week and were asked for a phone number for follow-up. Results could only be collected from the site of sample collection so could only take place at seven-day intervals from sample collection (as CHIEDZA is a weekly service). To maintain confidentiality, clients were asked for a pseudonym and samples were processed using an ID number. All clients with a positive test result who did not receive their test results on the same day were actively followed-up by telephone. Clients confirmed their identity by confirming their pseudonym. No results were disclosed by phone and clients were advised to attend to collect their results. Repeat phone calls were made weekly up to 2 months before a client was considered LTFU. Positive cases were treated according to national guidelines [33]. Individuals who reported STI symptoms at presentation and were found to have an STI syndrome were treated according to national guidelines for syndromic management [33]. If treatment was given on the day of presentation, they were not actively contacted about a subsequent positive CT/NG test result. Partner notification (PN) slips were offered to those who were treated for an STI, and all partners were offered presumptive treatment regardless of age and whether they resided in the intervention cluster.

### Data collection

Quantitative data on the CT/NG diagnostic cascade was collected between 25th November 2019 and 18th March 2020, including uptake of testing, proportion of samples processed on-site, number of days between sample collection and sample processing, number of days between sample collection and collection of results, proportion of clients collecting results and proportion of clients receiving treatment. Additionally, the GeneXpert error log was reviewed, and the number of error codes and samples affected was recorded.

One-off in-depth interviews were conducted with all nine CHIEDZA providers employed at time of interviews (four CHWs, two nurses, two youth workers, and one counsellor). Three further interviews were also conducted with team members involved in the development and co-ordination of on-site CT/NG testing: the trial co-ordinator and a microbiologist and a laboratory technician who developed the SOPs and conducted the training. A topic guide for the interviews was developed for the study (see additional file 1). The interviews investigated views on providing STI services; the use of the GeneXpert platform to provide point-of-care testing and the impact on their role, workload, and flow of clients; and perceptions on the effect of point-of-care CT/NG testing on clients. Interviews were conducted by three interviewers trained in qualitative interviewing and the particular topic guide: CM (female), PN (female) and KM (male) not involved in service delivery, in either English (*n* = 8) or Ndebele (*n* = 4). Participants were approached either during study team meetings or by phone and asked to participate in the interview. They were aware that the interviews were conducted by members of the CHIEDZA process evaluation team, in order to assess the implementation of the GeneXpert platform into services. Depending on travel and social distancing restrictions to prevent transmission of COVID-19 in place at the time of the interview, interviews were conducted by telephone (*n* = 5), video conference call (*n* = 3), or in person with social distancing and other infection prevention control measures observed (*n* = 4), with only the researcher and participant present. Seven of the interviews lasted between 19 and 39 min. The remaining five interviews on STI services were embedded within longer interviews as part of a broader evaluation of CHIEDZA services, each lasting between 60 and 129 min. All interviews were audio-recorded and translated and transcribed verbatim. Transcripts were not returned to participants, given the providers workloads, but the providers were included in the ongoing trial discussions, including on process evaluation findings.

### Data analysis

STATA version 16.0 (StataCorp, Texas, USA) was used for the quantitative data analysis. Categorical variables were described using frequencies and percentages. Continuous variables were described using either mean (standard deviation) or median (interquartile range) for normally distributed and non-normally distributed data, respectively. Treatment rates between samples processed on- and off-site were compared using Pearson’s chi-squared test.

Qualitative data were analysed using thematic analysis. Following familiarisation with data, initial coding was performed by KM (academic clinician) and reviewed by CMY (qualitative researcher). An inductive approach was used to develop themes, which were reviewed, named and defined [34]. NVivo 12 (QSR International) was used to assist with coding transcripts. Coding and themes were iteratively reviewed and refined, with thematic discussions involving KM, CMY, CM and PN. Data saturation was reached during analysis. Due to the small number of CHIEDZA providers working at the Bulawayo sites, quotes have not been attributed to a particular role of a participant to maintain confidentiality. Qualitative data has been reported according to the consolidated criteria for reporting qualitative research (COREQ) (Additional file 2) [35].

## Results

### CT/NG diagnostic Cascade

Between 25th November 2019 and 18th March 2020, there were 3426 attendances by 2335 clients at the four clusters. Uptake of testing was 40.3% (941/2335; 95% confidence interval (CI) 38.3–42.3%). The CT/NG prevalence among those tested was 19.9% (186/936; 95% CI 17.4–22.6%); results were not available for five clients. There were 158 (16.9%) tests positive for CT and 45 (4.8%) positive for NG. Seventeen (1.8%) were positive for both CT and NG.

**Fig. 2:**
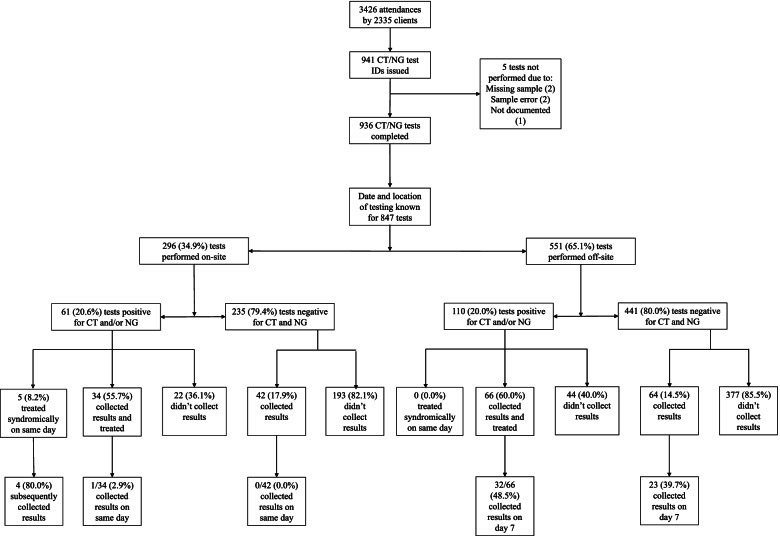
Flow chart for testing and treatment of CT/NG infections

Data on the date and location of testing was available for 847 tests. The full testing cascade is shown in Fig. 2, and the weekly breakdown on testing numbers is shown in Table 1. Out of 847 tests, 296 (34.9%) tests were performed on-site on the same day, 524 (61.9%) were performed off-site in Bulawayo, and 27 (3.2%) were sent to Harare for testing in early/mid-January due to the number of samples outstripping testing capacity. Of the 551 samples processed off-site, 400 (72.6%) were processed the day after collection, 107 (19.4%) were processed 2 days after collection, and 44 (7.9%) were processed three or more days after collection. There was a median 1 day (IQR 1–2) between collection and sample processing for samples processed off-site.

**Table 1:** Number of samples tested for CT/NG by week, disaggregated by site of testing

Week starting	Testing location, n (%)		Missing location	Total tested
	On-site	Off-site		
Two-module machine				
25-Nov-2019	8 (34.8%)	15 (65.2%)	2	25
02-Dec-2019	9 (39.1%)	14 (60.9%)	1	24
09-Dec-2019	3 (9.7%)	28 (90.3%)	0	31
16-Dec-2019	15 (21.4%)	55 (78.6%)	0	70
06-Jan-2020	14 (18.7%)	61 (81.3%)	0	75
13-Jan-2020	12 (24.5%)	37 (75.5%)	33	82
20-Jan-2020	7 (13.2%)	46 (86.8%)	20	73
27-Jan-2020	12 (29.3%)	29 (70.7%)	30	71
Two-module machine replaced by four-module machine on 04-Feb-2020				
03-Feb-2020	10 (17.2%)	48 (82.8%)	1	59
10-Feb-2020	41 (73.2%)	15 (26.8%)	0	56
17-Feb-2020	34 (47.9%)	37 (52.1%)	0	71
24-Feb-2020	44 (67.7%)	21 (32.3%)	2	67
02-Mar-2020	41 (51.3%)	39 (48.8%)	0	80
09-Mar-2020	33 (42.9%)	44 (57.1%)	0	77
16-Mar-2020	13 (17.3%)	62 (82.7%)	0	75
Total	296 (34.9%)	551 (65.1%)	89	936

Of the tests performed on-site, 61 (20.6%) were positive for CT/NG, of which a total of 39 (63.9%) received treatment: 8.2% (5/61) who were treated syndromically on the day of presentation; 1.6% (1/61) who collected their results and were treated on the same day as sample collection; and 54.1% (33/61) who presented subsequently for treatment. Of the 235 tests performed on-site that were negative for CT/NG, 42 (17.9%) clients collected their results, but none on the same day (Table 2).

**Table 2:** CT/NG prevalence, treatment, and results collection comparing on- and off-site testing (*N* = 847)

	On-site testing (*N* = 296)	Off-site testing (*N* = 551)
CT/NG Prevalence, n (%)	61/296 (20.6%)	110/551 (20.0%)
Proportion of positive results treated, n (%)	39/61 (63.9%)	66/110 (60.0%)
-Treated by syndromic management	5/61 (8.2%)	0/110 (0.0%)
-Xpert diagnosis and treatment on day of sample collection	1/61 (1.6%)	0/110 (0.0%)
-Xpert diagnosis with treatment subsequent to day of sample collection	33/61 (54.1%)	66/110 (60.0%)
Number of days to collect positive result, median (IQR)	14 (7–35)	14 (7–52.5)
Proportion of negative results collected, n (%)	42/235 (17.9%)	64/441 (14.5%)
-Results collected on day of sample collection	0/235 (0.0%)	–

Of the tests processed off-site, 110 (20.0%) were positive for CT/NG, of which 66 (60.0%) received treatment. None were treated syndromically, and so they had to return for treatment on a subsequent visit. Of those with negative results, 14.5% (64/441) subsequently collected their results. There was no significant difference in the proportion of those with positive test results who were treated whether tested on or off-site (64% (39/61) vs 60% (66/110); *p* = 0.61). There was also no difference in the median number of days taken to collect results for those with a positive result for tests performed on- and off-site (14 days (IQR 7–35) for on-site vs 14 days (IQR 7–52.5) for off-site testing).

Overall, a median 15.5 (IQR 11–20; range 2–59) tests were requested per day over the study period. The median (IQR) number of tests requested per day was 13.5 (8–19.25) and 17 (15–22.25) before and after the introduction of the four-modular device, respectively. Following the replacement, the proportion of samples processed on-site increased from 23.1% (86/373) to 44.3% (210/474).

### Error log

The error log revealed 60 error codes affecting 36 samples over the course of the study. In each instance, the sample was re-run, and 35 samples were subsequently successfully processed. Of note, 11 error codes were due to power supply or connector cable failure. A further 8 of these error codes represented connection problems resulting from a faulty ethernet cable connecting the GeneXpert machine and the laptop, with clustering of codes in early December. This was remedied after the providers discussed this with the laboratory technicians in Harare.

### Facilitators and barriers to provision of on-site CT/NG testing

The interviews with service providers revealed four themes related to the provision of same day CT/NG results associated with the i) Limitations of the diagnostic device, ii) Challenges with implementation in a community setting, iii) Provider buy-in, and iv) Competing priorities for clients. Potential solutions were also discussed. See Table 3 for sub-themes and supporting quotes.

**Table 3:** Themes and supporting quotes from interviews

Themes	Quote
***Limitations of the diagnostic device***	
**Analytic capacity**	**-***“That was never a success because at first, we had a machine that could run 2 samples then we got another which could run 4 samples and because there was a lot of samples to be done and other competing activities it was difficult to offer same day results.”* **-***“We collect samples let’s say 35 as an example, each sample takes about an hour to run and then we have 35 samples like how many hours do we have in a day of operating at the field? So it will mean that on a daily basis you will be able to run maybe 15 samples meaning that today’s samples won’t all be run there are some which are going to be left out. Then the next day it means we are going to start with the ones remaining from yesterday because there are expiration dates to these things. So you will find out that it was very impossible to give clients their results on daily basis, it was just impossible because of the turnaround time and all”* **-***“So the following day you would start with yesterday’s remainder and you had the load for that day and sometimes it would even flow over to the third day which means that it needed 3 days to maintain the time and running those tests for clients so that the samples wouldn’t expire as well.”* **-***“Then the other thing is that we were getting more samples than what we had initially anticipated, we had more samples than what the machine could handle. Initially we had planned that a person would collect the sample, the test is run, and they get the results the same day, but we were getting more samples than machines. Also, the test takes about 90 min. We ended up having a backlog of samples and people were not able to get their samples on the same day. CHIEDZA operates from Monday to Thursday then on a Friday it’s a debrief day, so we decided to run the samples on a Friday. We leave the office early and go to the community early to start doing the running of the samples. Also, before going to the field everyday someone got to the office early and started running the samples before actually going to the field. Then later on towards the end of the study we got another bigger machine and it helped to reduce the pressure.”* **-***“I think if we can get a bigger machine that can run more cartridges at the same time it will be better. When we started we had a machine that ran two then they brought another one that could run four samples which was better. A bigger machine will help to ensure that we have a smaller backlog.”*
**Analytical errors**	**-***“But then the thing about that machine is that it is very sensitive to a lot of things because you will find out that you would have placed a sample in there to run only to realize after 45 min that there is an error maybe a bubble entered the sample, or the machine overheated or for whatever reason it will jam on you or give you an error after having been confident that I have done a proper thing yet there is a problem. But with time what I noticed is that we were able to use it but during the first days we didn’t do things properly but with experience and time we were able to use it properly”* **-***“Yes, so you will just be managing the booths and the machine and that meant that it was one person doing two jobs and that may bring out errors but then we ended up doing that trying to make up for that”* **-***“I think the process of moving from the test tube to the cartridge, so that there are no bubbles and the correct measurement, at times you will be in a rush to do more samples and you end up missing the correct measurement and having bubbles.”* **-***“There was a faulty cable which did not work properly. We ended up losing some samples because of that. At first, we did not know what the problem was we later discovered it was the faulty cable. The service providers of course some were confident with the machine whilst others were not so confident but all of them were trained. We had errors here and there especially at the start when we were starting because of the faulty cable but with time it got better.”*
***Challenges with implementation in a community setting***	
**Unstable electrical supply**	**-***“And when there is an electricity issues you need to have a backup generator on standby.”* **-***“The main problem was the electricity load shedding. When the battery had small power and there was no electricity it resulted in a backlog of sample testing.”* **-***“The other thing when we started, we had power cuts which created challenges. Eventually we got a generator but we did not have the technical know-how of how the machine responded to sudden change of power supply from Zesa to generator and if we switched the running samples automatically gave error messages and this gave us pressure in terms of backlog of specimen since we had to rerun all over again.”* **-***“The GeneXpert machines are very sensitive to electricity changes, so if there is a power cut or a small shift in power supply it just aborts the running process and it means that you to start afresh and you could lose your cartridges. All this has financial implications. In Bulawayo they did not have many electrical issues in their sites, they had back up power batteries which then enabled them to have more testing time in case there were some power cuts.”* **-***“We also had the problem of electricity when it goes and comes back that sudden change means you have to start the samples all over again.”*
**Non-controlled conditions**	**-***“At [site name], there is no shelter it is just an open space affected by weather elements and I do not think that is ok.”* **-***“It was ok except for the [site name] site during the rainy season, those tents are porous and it affected our operations on such days are seriously affected.”* **-***“We had to monitor the temperature of the cartridges, samples and the machine. At some point we bought a thermometer, and we had a temperature log. There were concerns about the temperatures going up in the office during the day.”*
**Inadequate space**	**-***“The fifth booth was for the machine; it was also the same booth that the nurses used for checking out and treating STIs so when they were busy inside you had to wait for them. So, the space was very limited.”* **-***“The space was not enough, we had a tent where we put all equipment and that is the tent we used for the running of samples, at times the urine will spill, and you have to disinfect the area but there is congestion all over.”* **-***“It helps to do our testing in a private setup. Because we were using the nurse’s lab to do these tests of which if a person wants to come and change a specimen, they then disturb a nurse who will be working on the lab so an extra tent will be good.”*
**Challenges associated with moving machine between sites**	**-***“The other challenge is setting up the machine and folding everything back after use every day.”* **-***“And also, the running around with the samples, imagine with the urine and also storage of the urine samples that was another challenge and also considering that we had one car for the staff, for the luggage, for the tents and those machines are delicate and so that was another challenge when we were the ones doing it; and especially moving with the gadgets to and from I think it reduces the lifespan of the machine itself.”* **-***“If we had a place where they are just placed let’s say we would run the samples and then tomorrow we would bring another set of samples and they would be taken to a place where they are being run specifically there and before the end of day, I think that would have been a better option to have a central place where the tests are all being run. It’s a bit risky travelling with the machine and we actually gave the machine a nickname it’s called Precious and after some travelling, we would ask like is Precious sitting very well? Is Precious safe? Because it was our precious gadget you know.”* **-***“There is a difference because in the lab you do not move the machine frequently once its set it stays in that position for a long time, so you do not expect any hardware or connection errors. I guess when you are moving the machine around connected to batteries, when the batteries lose power, and you want to switch to electricity you are bound to lose that run.”*
***Provider buy-in***	
**Training**	**-***“The training we got was enough for me because the machine is straightforward, and it does not need a lot of work … I think everything was covered during the training.”* **-***“No, it was very simple because we received some training before we started running the machine. Any complications, I asked the nurses for assistance.”* **-***“I would say the training was 50% adequate because it was a one-day training, of course it was a practical training but the fact that it was just one day I could not grasp all the concepts at once.”* **-***“As much as all the staff members were trained on using the machine some of them did not have the lab background and it was difficult for them to adhere to the correct procedures of handling specimen. It came down to us the nurses doing all the specimen with the help of (name), so she was ok with lab things. The other staff members just helped with handling specimen, all this gave us pressure because we had to do all the work and chase the targets in booths as well.”*
**Increased workload and impact on other services**	**-***“The queues were moving slow because you had to wait for one client and finish with them to avoid a mix-up of samples. If a client took the container and took a long time to bring a sample back, you had to wait for them until they got back. In the meantime, the queues would be growing longer.”* **-***“Roles for everyone changed … We were now laboratory scientists/ community health workers / youth worker/ lab scientists. Remember our role was to collect samples in the booth like we were the point of collection by asking clients to give us their samples. But tomorrow again you will be collecting samples depending on whether you are in the booth at that time running the laboratory at the same time. So we would run our own tests, and we would also collect; the roster was there already notifying us who is on the duty.”* **-***“You would find out that if you didn’t manage to finish running the tests if it’s your duty, you would have to go to the office on Friday if it’s your duty and even on weekends if you don’t manage to finish again. On Saturdays I remember we had a duty roster, and everyone was aware of their own Saturday to come to work so our roles definitely changed and the workload increased too.”* **-***“Remember CHIEDZA is too much multi-faceted, you are a construction worker in the morning you are supposed to pitch the tents and they are very heavy; you work in the afternoon and when it’s knock off time you unpitch those tents again. And then let’s say you were the one on duty of collecting urine samples and running them, and the GeneXpert machine has set time for switching on and off and then you have to record the results. You cannot expect someone else to come and record the results for you, so you record on the tablets and input on the forms so definitely our roles changed and workload increased”* **-***“Yes we can say we had more work to do yes because almost everyone would be in the booth of course and we would exchange after maybe 4 h then we change the other team to go into the booth as well. But then we needed to allocate at least one person to be running the GeneXpert machine during the day and that meant that we would one or two people less for the day in the booths.”* **-***“[In the Harare lab] There was more workload to do. It required that we work over the weekends to cover more ground, for example I could have 4 runs for Harare and maybe 2 or 3 more runs for Bulawayo and each run is about 90 min and we did not have a lot of machines. So, it really meant trying to create more time and it ended up in a situation where we had a backlog.”* **-***“Number 1, we need someone who will be responsible for the testing part like the running of samples because having one person taken out of the team manning that machine would mean that we are one man down and we are incapacitated. So at least if we know that we have a team member who is solely responsible for the running of the tests then it will be fine. Whether they have finished running the tests or not it will be entirely up to them to know how to run them as per their order it will be much easier because as a community health worker I will know that I am responsible only for collecting the samples and giving them to that person that would be great you know having that feeling that there is someone there who is manning the booth for samples only.”*
**Providers not offering a same day testing service**	**-***“So, what we did was to inform all the clients to come back the following week to get their results whether positive or negative. We separated the positive results from the negative ones because we were most interested in the positive cases. If they did not come the following week, we then phoned them about their results and advised them to come back with their partners.”* **-***“No, I don’t recall any who did that, specifically for me I did not give results same day.”* **-***“So we would like; run the test this week and then you get your results next week through text messages telling the clients that their results are ready.”* **-***“No we did not, because we explained to them that we could only give results after a day or two.”* **-***“Almost everyone would just come back because the machine takes 90 min so you can’t ask a client to be waiting for such a long time at the site so because of the backlog we would end up running samples that were collected on Monday maybe on Wednesday after about 7 maybe after about more than 48 h so you then need to call the clients to come and get their results or send text messages to just say we are notifying you that your results are out, can you come and present yourself at our site for result collection.”*
***Clients***	
**Clients’ competing priorities**	**-***“I think for them it will not be a priority, some just do the test just because it is being offered and when you call them to come and get the results, they are just not bothered about coming to get them.”* **-***“Uhm, when using the GCCT screening machines I think the challenge is on follow-up of those clients to come and get their results because some of them just come to get tested and they don’t come back for their results.”* **-***“Most of them were saying that they are busy they need to rush somewhere though I realised that they do not want their results same day because of fear.”* **-***“When the results came out positive it became a big challenge because most clients put up fake contact details and so we could not contact them. You call that number it does not go through, because it was my duty to do contact tracing, you try again the following day, but the number will still not go through. Then we had to go back to their houses to look for them, it was really hard.”* **-***“Most of them were saying they cannot wait for 90 min they will rather go home and come back the following week.”* **-***“I think it will be difficult for youths to do that because already for them to wait for 20 min for HIV test results is a challenge, even waiting for 5 min they do not want these young people are always in a hurry. I do not see how they will sit around and wait for 90 min, maybe one or two will wait but the rest will not. A better option could be that they go back home then they come back to get the results after an hour or so.”* **-***“Then there were clients who came early morning, and they want their results at the same time. When you explained to them the 90 min waiting period, they did not want to understand that.”* **-***“No, they did not wait for their results, most of them did not want to wait for their results, those who came very early in the morning we told them to come for their results before 5 pm on that day. The rest came the following week or waited for the following week to collect their results.”* **-***“Only one or two boys who had come early and whilst their samples were running, they were playing pool in the social area.* **-***Yes, we had some but only like if they tell you that I will be going to school especially the University students who would tell you that next week I will be going back to school and then you will be like it’s okay you can still come back after two months. We would also try to run their tests first and then wait for the results that’s if they are willing to wait for 2 h and that would also depend on what time they would come.”* **-***“The test takes 90 min, and it is a long time for anyone to wait. A person spends 30 min in the booth and then they have to wait for 60 min for their results, a lot of people did not want to wait, and they did not wait.”*

#### Limitations of the diagnostic device

##### Analytic capacity

Despite an increase in testing capacity by replacing the two-module instrument with a four-module instrument, providers noted that sample throughput over the six and a half hours available for testing was insufficient, resulting in a backlog of samples which needed to be tested off-site. As a result, samples were processed the following day or later and so “*it was difficult to offer same day results.”*

*“The issue of same day results is a bit tricky because the big machine runs 4 samples in 90 minutes, so what it then means is that if we spent 5 hours on site, only about 12 samples will be done before we close maybe out of a possible 30 samples that would have been collected.”*

##### Analytical errors

Sample runs resulting in an error message were noted to have caused disruption for CHIEDZA services. Once a sample was loaded onto the instrument, providers usually left the instrument to continue service provision. Hence errors went unnoticed until the provider returned at the end of the sample run. Re-running the sample with an erroneous result delayed the testing of other samples. This in turn reduced the number of samples analysed on site each day. Providers noted that error rates reduced *“with experience and time”* as they became more confident using the device.

#### Challenges with implementation in a community setting

The community outreach model of service delivery meant that the diagnostic instrument had to be unpacked and installed on a daily basis. Concerns were raised about transporting the instrument between sites every day. This included the extra work required to move the machine, as well as the potential “*risk travelling with the machine*”, which the providers considered a “*precious gadget*”.

Reported challenges included electrical supply, high temperatures and weather conditions such as wind and rain. This was particularly challenging at one site, where services were provided in an outside space rather than inside a community centre.

*“We did not work in a lab setting where the environment is controlled. The machine needs to operate in cool temperatures; it does not want inconsistencies like noise, increase in temperature and so on, so you can imagine a site like [site name] you will be in trouble.”*

Furthermore, due to shortage of space and for safety the instrument was placed in one of the booths, which was simultaneously used by nurses for clinical consultations. This sometimes resulted in sample processing and testing being delayed, as “*when they were busy inside you had to wait for them”.*

#### Provider buy-in

##### Training

Training was an important facilitator ensuring that providers were able to use the GeneXpert machine. The training received was generally felt to be adequate and most providers found using the machine *“simple and straightforward”*. Some providers noted that they did find using the machine difficult initially, however with experience they became more confident in running tests.

##### Increased workload and impact on other services

The introduction of on-site CT/NG testing led to an increase in workload for CHIEDZA providers. This included both a higher intensity of work at the sites as providers had to juggle CT/NG testing with other duties, as well as the need for excess samples to be processed outside of normal working hours.

*“What it meant was that a person had to be in the booth and at the same time run the test, this put everyone under unnecessary pressure and the number of samples kept increasing whilst people were doing exchanges in the booths. We ended up being forced to run the samples over the weekend and we developed a roster to run these samples over the weekend.”*

##### Providers not offering a same day testing service

Some providers did not view on-site GeneXpert testing as providing same day results and did not think it was feasible to do so. When results were given out on the same day as sample collection, this was seen as an exception rather than the rule. Providers generally informed clients to return the following week to collect their results.

*“They would leave the samples and be told to come back next week, but you will find out that because of that some of the clients up to now they haven’t come back to collect their results.”*

#### Competing priorities for clients

There was a strong perception amongst providers that there was limited demand from clients for same day results, given the time clients had to wait, except in specific limited circumstances. Providers perceived clients’ choice to wait for their results to be influenced by their level of interest in the results, worry surrounding a potential positive result and the time commitment to receive results. There was a perception that clients simply *“cannot wait for 90 minutes”* for their results. Clients who did receive same day results were noted to either spend time in the social area or go home and return.

*“Most clients did not want to wait for their results they just said they will leave their details and they will collect next time when they come. They were few who were willing to wait, and it was mainly boys who then went to the social area to play but no girls waited for their results. They said they had to go back home and do other chores that needed to be done.”*

## Discussion

This study has demonstrated some of the challenges and limitations associated with on-site CT/NG testing for youth in community-based settings, using the GeneXpert platform. Our operational environment was challenging with an intermittent electricity supply, and testing performed in a non-laboratory environment by staff who were not laboratory technicians. Despite this, we have demonstrated the feasibility of processing samples on-site in such an environment. Furthermore, the number of error codes was low and crucially the providers generally felt that their training was adequate and became more confident with time. Of note, feasibility of GeneXpert as part of a mobile testing unit has previously been shown for tuberculosis screening [36]. However, as presented in the results, several key barriers prevented an operational same-day testing and treatment strategy from being implemented.

We reported a previous study also nested within the CHIEDZA trial that investigated uptake of STI testing in youth, which showed that only two-thirds of those with positive CT/NG tests were treated, the remainder being LTFU. In that study, STI testing was performed in a central laboratory with results available in a week [3]. The main potential benefit of on-site testing is the possibility of providing results on the same day thus reducing attrition between testing and treatment. However, only one client collected their test result on the same day. This likely contributed to the lack of a significant difference in the proportion of clients treated between those with samples tested on-site versus off-site.

While other studies in resource-limited settings have demonstrated the feasibility of offering same day results and treatment using the Xpert® CT/NG [23, 25, 28], this study found that there were major challenges in a non-clinical setting. Same-day testing and treatment has been incorporated in research surveys of high-risk population groups, for example in men who have sex with men, transgender women, and female sex workers in Papua New Guinea [28]. However, as the purpose of sampling was for a survey, this may not accurately represent a service provision situation. In a clinical context, Xpert® CT/NG testing has been offered in antenatal care, with the majority of women with positive results receiving same day treatment [23, 25]. However, as the youth clients in this study were accessing a range of services (of which STI screening was one) in a non-clinical setting, they may have had less drive to access results as other groups, such as pregnant women. Additionally, the flow of clients and other activities required differ between this study and antenatal clinics, making comparison difficult.

The barriers to on-site STI testing were multifactorial and highlight the importance of understanding the local context in the design of interventions. Less than a quarter of samples were processed on-site using the two-module device. As a result, samples not processed on the same day had to be processed during the following days. This in turn affected testing capacity for the remainder of the week, hindering the ability to provide same day results for clients. Increased testing capacity led to 20% more samples being processed on-site. However, a larger machine is more difficult to transfer between sites. Testing capacity could potentially be optimised through risk stratification of clients, to either only test higher risk individuals, or to prioritise sample processing. However, if high prevalence is found amongst individuals without risk factors, such as were found amongst young people in Harare, then such risk prediction tools will be insufficiently sensitive [37].

Even with increased capacity, the 90-min analytic time was still prohibitive to providing same day results to many young people. Providers highlighted that young people often had competing priorities and were generally unwilling to wait for results. Waiting time is a frequently mentioned barrier to youth accessing health services in resource-limited settings, and young people may be less willing to wait than adults for test results [29]. Importantly, CHIEDZA services have been co-designed with young people to be “youth-friendly”, with music and activities, and where clients can spend time with their friends whilst they wait for their results. Therefore, the apparent unwillingness to wait a minimum of ninety minutes in a setting such as this, may be exaggerated in conventional clinical settings that may be less acceptable to youth [29].

There are a number of point-of-care tests for CT and NG in the pipeline with shorter analytic times [38]. Their development and use may make it more feasible to provide results and to treat clients on the same day. A study among university students in the United States found that 83% were willing to wait for their CT/NG results using the binx Health *io* assay (binx Health Ltd., Trowbridge, UK), which has an analytic time of thirty minutes [39]. Even greater flexibility could be allowed if a successful lateral flow test for CT/NG could be developed. The ten-minute analytic time and ease of use of the OSOM® Trichomonas Rapid Test (Sekisui Diagnostics, Bedford, Massachussets, USA) has allowed self- and home-testing to be acceptable and feasible testing strategies [40, 41].

To our knowledge, this is the first study to evaluate the implementation of the GeneXpert platform for on-site STI testing for youth in a non-clinical, community-based setting in sub-Saharan Africa. This study used mixed methods enabling exploration of issues related to STI testing in both breadth and depth. We interviewed each member of the service provider team including the team that developed the point-of-care testing and collected detailed data on the diagnostic cascade. We acknowledge several limitations. We did not interview clients, and so we can only infer their perceptions from themes elicited from CHIEDZA study personnel and providers. As a result, although waiting time for clients is undoubtedly a barrier, its perceived relative influence on the feasibility of providing on-site testing is potentially skewed. We must also consider that testing was only performed for chlamydia and gonorrhoea, and so the results may not reflect some of the additional barriers that may become apparent if offering testing for other common STIs. Data on testing date and location was missing for 89 samples and recording of sample errors required providers to document an error on a log. Given the busy service, this may not always have happened and so the 60 errors that occurred may be an underestimate.

## Conclusion

Syndromic management is insufficient for STI control as asymptomatic infections cannot be detected and treated. While mobile diagnostic platforms for CT/NG testing have been developed, analytic capacity and testing time are barriers to their use as a true point-of-care test. Some of these barriers are likely to be addressed as newer platforms are developed that meet the ASSURED criteria. More generally, approaches to improve access to results and linkage to treatment are needed, particularly in youth who have high burdens of STIs, in order to develop strategies that are scalable. As shown with other conditions such as HIV and TB, strategies for delivery of testing and treatment must be diverse, nuanced, and take account of the local context.

## Supplementary Information

**Additional file 1.** Interview topic guide.

**Additional file 2.**

## Data Availability

The datasets used and/or analysed during the current study are available from the corresponding author on reasonable request. Additionally, individual, anonymised participant data and a data dictionary will be available through The London School of Hygiene & Tropical Medicine repository (Data Compass) 12 months after publication of results.

## References

[1] Rowley J, Vander Hoorn S, Korenromp E, Low N, Unemo M, Abu-Raddad LJ (2019). Chlamydia, gonorrhoea, trichomoniasis and syphilis: global prevalence and incidence estimates, 2016. Bull World Health Organ.

[2] Torrone EA, Morrison CS, Chen PL, Kwok C, Francis SC, Hayes RJ (2018). Prevalence of sexually transmitted infections and bacterial vaginosis among women in sub-Saharan Africa: an individual participant data meta-analysis of 18 HIV prevention studies. PLoS Med.

[3] Martin K, Olaru ID, Buwu N, Bandason T, Marks M, Dauya E (2021). Uptake of and factors associated with testing for sexually transmitted infections in community-based settings among youth in Zimbabwe: a mixed-methods study. Lancet Child Adolesc Health.

[4] Fleming DT, Wasserheit JN (1999). From epidemiological synergy to public health policy and practice: the contribution of other sexually transmitted diseases to sexual transmission of HIV infection. Sex Transm Infect.

[5] World Health Organization (2004). Guidelines for the management of sexually transmitted infections.

[6] Farley TA, Cohen DA, Elkins W (2003). Asymptomatic sexually transmitted diseases: the case for screening. Prev Med.

[7] Der JB, Grint D, Narh CT, Bonsu F, Grant AD (2020). Where are patients missed in the tuberculosis diagnostic cascade? A prospective cohort study in Ghana. PLoS One.

[8] Tang EC, Segura ER, Clark JL, Sanchez J, Lama JR (2015). The syphilis care cascade: tracking the course of care after screening positive among men and transgender women who have sex with men in Lima, Peru. BMJ Open.

[9] Swartzendruber A, Steiner RJ, Adler MR, Kamb ML, Newman LM (2015). Introduction of rapid syphilis testing in antenatal care: a systematic review of the impact on HIV and syphilis testing uptake and coverage. Int J Gynaecol Obstet.

[10] Munkhuu B, Liabsuetrakul T, Chongsuvivatwong V, McNeil E, Janchiv R (2009). One-stop service for antenatal syphilis screening and prevention of congenital syphilis in Ulaanbaatar, Mongolia: a cluster randomized trial. Sex Transm Dis.

[11] Allan-Blitz L-T, Herrera MC, Calvo GM, Vargas SK, Caceres CF, Klausner JD (2019). Venue-based HIV-testing: an effective screening strategy for high-risk populations in Lima, Peru. AIDS Behav.

[12] Bien CH, Muessig KE, Lee R, Lo EJ, Yang LG, Yang B (2015). HIV and syphilis testing preferences among men who have sex with men in South China:a qualitative analysis to inform sexual health services. PLoS One.

[13] Nkamba D, Mwenechanya M, Kilonga AM, Cafferata ML, Berrueta AM, Mazzoni A (2017). Barriers and facilitators to the implementation of antenatal syphilis screening and treatment for the prevention of congenital syphilis in the Democratic Republic of Congo and Zambia: results of qualitative formative research. BMC Health Serv Res.

[14] Ruffinen CZ, Sabidó M, Díaz-Bermúdez XP, Lacerda M, Mabey D, Peeling RW (2015). Point-of-care screening for syphilis and HIV in the borderlands: challenges in implementation in the Brazilian Amazon. BMC Health Serv Res.

[15] Mabey DC, Sollis KA, Kelly HA, Benzaken AS, Bitarakwate E, Changalucha J (2012). Point-of-care tests to strengthen health systems and save newborn lives: the case of syphilis. PLoS Med.

[16] Lipsitz MC, Segura ER, Castro JL, Smith E, Medrano C, Clark JL (2014). Bringing testing to the people - benefits of mobile unit HIV/syphilis testing in Lima, Peru, 2007-2009. Int J STD AIDS.

[17] Verwijs MC, Agaba SK, Sumanyi JC, Umulisa MM, Mwambarangwe L, Musengamana V (2019). Targeted point-of-care testing compared with syndromic management of urogenital infections in women (WISH): a cross-sectional screening and diagnostic accuracy study. Lancet Infect Dis.

[18] Peeling RW, Holmes KK, Mabey D, Ronald A (2006). Rapid tests for sexually transmitted infections (STIs): the way forward. Sex Transm Infect.

[19] Murtagh MM (2019). The point-of-care diagnostic landscape for sexually transmitted infections (STIs).

[20] Xie T-A, Liu Y-L, Meng R-C, Liu X-S, Fang K-Y, Deng S-T (2020). Evaluation of the diagnostic efficacy of Xpert CT/NG for Chlamydia trachomatis and Neisseria gonorrhoeae. Biomed Res Int.

[21] Cazabon D, Pande T, Kik S, Van Gemert W, Sohn H, Denkinger C (2018). Market penetration of Xpert MTB/RIF in high tuberculosis burden countries: a trend analysis from 2014 - 2016. Gates Open Res.

[22] Gadoth A, Shannon CL, Hoff NA, Mvumbi G, Musene K, Okitolonda-Wemakoy E (2020). Prenatal chlamydial, gonococcal, and trichomonal screening in the Democratic Republic of Congo for case detection and management. Int J STD AIDS.

[23] Badman SG, Vallely LM, Toliman P, Kariwiga G, Lote B, Pomat W (2016). A novel point-of-care testing strategy for sexually transmitted infections among pregnant women in high-burden settings: results of a feasibility study in Papua New Guinea. BMC Infect Dis.

[24] Wynn A, Ramogola-Masire D, Gaolebale P, Moshashane N, Agatha Offorjebe O, Arena K (2016). Acceptability and feasibility of sexually transmitted infection testing and treatment among pregnant women in Gaborone, Botswana, 2015. Biomed Res Int.

[25] Morikawa E, Mudau M, Olivier D, de Vos L, Joseph Davey D, Price C (2018). Acceptability and feasibility of integrating point-of-care diagnostic testing of sexually transmitted infections into a south African antenatal care program for HIV-infected pregnant women. Infect Dis Obstet Gynecol.

[26] Garrett NJ, Osman F, Maharaj B, Naicker N, Gibbs A, Norman E (2018). Beyond syndromic management: opportunities for diagnosis-based treatment of sexually transmitted infections in low- and middle-income countries. PLoS One.

[27] Stime KJ, Garrett N, Sookrajh Y, Dorward J, Dlamini N, Olowolagba A (2018). Clinic flow for STI, HIV, and TB patients in an urban infectious disease clinic offering point-of-care testing services in Durban. South Africa BMC Health Services Research.

[28] Badman SG, Willie B, Narokobi R, Gabuzzi J, Pekon S, Amos-Kuma A (2019). A diagnostic evaluation of a molecular assay used for testing and treating anorectal chlamydia and gonorrhoea infections at the point-of-care in Papua New Guinea. Clin Microbiol Infect.

[29] Newton-Levinson A, Leichliter JS, Chandra-Mouli V (2016). Sexually transmitted infection Services for Adolescents and Youth in Low- and middle-income countries: perceived and experienced barriers to accessing care. J Adolesc Health.

[30] Chikwari CD, Dringus S, Ferrand RA (2018). Barriers to, and emerging strategies for, HIV testing among adolescents in sub-Saharan Africa. Curr Opin HIV AIDS.

[31] Sanders EJ, Agutu CA, Graham SM (2021). Multiple HIV testing strategies are necessary to end AIDS. AIDS.

[32] Community based interventions to improve HIV outcomes in youth: a cluster randomised trial in Zimbabwe (CHIEDZA): London School of Hygiene & Tropical Medicine. 2019.

[33] Zimbabwe Ministry of Health and Child Care (2019). National STI Management Guidelines.

[34] Braun V, Clarke V (2006). Using thematic analysis in psychology. Qual Res Psychol.

[35] Tong A, Sainsbury P, Craig J (2007). Consolidated criteria for reporting qualitative research (COREQ): a 32-item checklist for interviews and focus groups. Int J Qual Health Care.

[36] Bassett IV, Forman LS, Govere S, Thulare H, Frank SC, Mhlongo B (2019). Test and treat TB: a pilot trial of GeneXpert MTB/RIF screening on a mobile HIV testing unit in South Africa. BMC Infect Dis.

[37] Kranzer K, Simms V, Dauya E, Olaru ID, Dziva Chikwari C, Martin K (2021). Identifying youth at high risk for sexually transmitted infections in community-based settings using a risk prediction tool: a validation study. BMC Infect Dis.

[38] Toskin I, Govender V, Blondeel K, Murtagh M, Unemo M, Zemouri C (2020). Call to action for health systems integration of point-of-care testing to mitigate the transmission and burden of sexually transmitted infections. Sex Transm Infect.

[39] Gettinger J, Van Wagoner N, Daniels B, Boutwell A, Van Der Pol B. Patients Are Willing to Wait for Rapid Sexually Transmitted Infection Results in a University Student Health Clinic. Sex Transm Dis. 2020;47(1). 10.1097/OLQ.0000000000001083 31856075

[40] Lippman SA, Jones HE, Luppi CG, Pinho AA, Veras MA, van de Wijgert JH (2007). Home-based self-sampling and self-testing for sexually transmitted infections: acceptable and feasible alternatives to provider-based screening in low-income women in São Paulo. Brazil Sex Transm Dis.

[41] Jones HE, Altini L, de Kock A, Young T, van de Wijgert JHHM (2007). Home-based versus clinic-based self-sampling and testing for sexually transmitted infections in Gugulethu, South Africa: randomised controlled trial. Sex Transm Infect.

