# Supplementary material for: “It was difficult to offer same day results”: evaluation of community-based point-of-care testing for sexually transmitted infections among youth using the GeneXpert platform in Zimbabwe
Source: BMC Health Serv Res. 2022 Feb 10;22:171. doi: 10.1186/s12913-022-07557-7 (PMC8830017; doi:10.1186/s12913-022-07557-7)
Supplement: Supplementary file 1 — Additional file 1. Interview topic guide. [file 12913_2022_7557_MOESM1_ESM.docx]

| We have two main areas that we would like to investigate in these interviews:   1. Topic 1: Experiences of providing STI services 2. Topic 2: Experiences of using on-site GeneXpert testing at CHIEDZA sites   The questions in this topic guide are not exhaustive and they are not prescriptive. This guide is to help you understand the primary areas of interest to pursue in the interviews. This guide should also give you some suggestions about how to word questions and approach the topics so that they may be likely to feel increasingly comfortable talking to you. They are just example questions. Ideally you should not take this guide into all your interviews, but make sure that you are familiar with it so that you can be responsive to what the participant is telling you through listening- but be confident that you are exploring the primary topics of interest. However, the most important point is to listen to what the young person is telling you and respond to that. Try to integrate pieces of information that they have told you into your questions at various points of the interview to demonstrate that you are concentrating and listening to what they are saying. You need to show that you have a compassionate curiosity to understand what it is that they are going through, what helps and what could be adapted to help them more. | | | |
| --- | --- | --- | --- |
| **Key areas of investigation** | **Rationale** | **Example questions** | **Explanatory notes** |
| **Topic 1: Experiences of providing STI services** | | | |
| **Reflections on offering STI services in the STI pilot last year** | STI screening was piloted last year within CHIEDZA and is going to be re-introduced in September/ October this year. We would like to learn from providers’ experiences of offering STI screening last year, and understand their perceptions on providing this service last year. | What was your experience of offering STI screening in the pilot last year?  How was STI screening integrated into the other CHIEDZA services?  What challenges did you encounter with STI screening?  How did you overcome these challenges?  Testing uptake increased over the course of the pilot. How was that achieved?  How do you think clients viewed STI screening?  Do you have any particular stories or experiences of offering STI screening that you would like to share? | Here it would be good to get at both the operational side of the service, as well as their personal experiences as providers. |
| **Reflections on offering STI testing for symptomatic clients in CHIEDZA** | Currently, in CHIEDZA (outside of the pilot last year) STI testing is offered, but only for clients with symptoms (syndromic management). We would like to understand their experiences of providing this service. | How does offering STI testing only for those with symptoms differ from the STI screening?  From your perspective, how have you found offering STI testing for symptomatic clients?  How do you think clients perceive this service? |  |
| **Suggestions for the re-introduction of STI screening in CHIEZA** | We would like to garner their ideas and suggestions of ways to improve STI services within CHIEDZA, in order to help us design STI screening services when they will be added to the CHIEDZA package of care. | What improvements would you suggest for better provision of STI screening within CHIEDZA?  How do you think uptake of testing could be improved?  How do you think linkage to treatment could be improved?  How do you think the partner notification process could be improved? | We need to recognise here that they may not have all the answers of having solutions to improve the STI screening services. |
| **Recommendations for feasibility, scalability, sustainability** |  | In your opinion, what could be done to make CHIEDZA better? FP/SRH services better |  |
| **Topic 2: Experiences of using on-site GeneXpert testing at CHIEDZA sites** | | | |
| **Use of the GeneXpert machine and impact on role** | In Harare and Mashonaland East, the GeneXpert machine for STI testing was in a central lab. However, at CHIEDZA sites in Bulawayo, the GeneXpert machine was situated on-site in the community centres. CHIEDZA providers managed the GeneXpert machine, allowing for same day results for some clients. We would like to learn from providers’ experiences in using the GeneXpert machine and incorporating it into their role at CHIEDZA. | Did your role change after STI screening was introduced at CHIEDZA?  What proportion of your time at CHIEDZA was spent using the GeneXpert machine?  How was your experience doing STI testing using the GeneXpert machine?  Were there any aspects of using the GeneXpert machine that you found difficult?  Did you feel adequately trained to use the GeneXpert machine?  Are there any aspects that you would have liked to have received more training or support on? |  |
| **Reflections on the effect of using the GeneXpert machine on workload and flow** | Providing on-site STI testing in community settings is currently a very unique situation in Zimbabwe. The experience in Bulawayo may help us learn how to incorporate point-of-care tests for STIs into healthcare services more generally as they become more widely available. We therefore want to understand from the providers’ perspective the effect of using the GeneXpert machine on both workload and client flow. | How did incorporating GeneXpert testing affect your workload?  How did it affect waiting times and flow of clients through CHIEDZA?  What did you think about the space available to provide GeneXpert testing at the CHIEDZA sites?  Has providing same day results for STI testing been successful at CHIEDZA? |  |
| **Perceptions on the effect of using the GeneXpert machine on clients** | Potentially having same day results is also likely to be a novel experience for clients attending CHIEDZA. We would therefore like to explore how the providers perceive the effect of on-site GeneXpert testing on clients. | Did many clients receive their results on the same day?  Did many clients wait for their results?  What did clients think about getting same day STI results at CHIEDZA?  Why do you think clients chose to wait for their STI results?  Why do you think clients chose not to wait for their STI results? |  |
| **Thoughts of the providers on how the provision of STI testing at CHIEDZA sites in Bulawayo differs from that in Harare or Mashonaland East** | As on-site testing is not provided in Harare or Mashonaland East but is provided in Bulawayo, we would like to hear the providers’ opinions on if they feel provision of on-site testing has been a positive or negative experience. | Do you feel that providing results on the same day as testing is advantageous?  How does it make you feel that same day STI results can be provided in Bulawayo but not at CHIEDZA sites in Harare or Mashonaland East?  What are the main challenges to providing on-site STI testing at CHIEDZA?  Would you change how STI testing has been implemented at CHIEDZA sites in Bulawayo? |  |

The interviewer should give space for the interviewee to add any further details that they want to add, or ask any questions.

The interviewer should say that we’ve come to the end of the interview. They should thank the interviewee for their willingness to talk and participate in this discussion. They should explain how the interview will be used, and reiterate that information that the interviewee has shared will inform CHIEDZA and wider research, and things they have said may be quoted, but that it will not be linked back to the particular interviewee.
